# Design and Implementation of an Architectural Framework for Web Portals in a Ubiquitous Pervasive Environment

**DOI:** 10.3390/s90705201

**Published:** 2009-07-02

**Authors:** Muhammad Taqi Raza, Seung-Wha Yoo, Ki-Hyung Kim, Seong-Soon Joo, Wun-Cheol Jeong

**Affiliations:** 1 USN Networking Research Team / Electronics and Telecommunications Research Institute (ETRI), South Korea; E-Mails: ssjoo@etri.re.kr (S.-S.J.); wjeong@etri.re.kr (W.-C.J.); 2 Information and Communication Department / Ajou University, South Korea; E-Mails: swyoo@ajou.ac.kr (S.-W.Y.); kkim86@ajou.ac.kr (K-H.K.)

**Keywords:** Ubiquitous era (U-era), web portal, Ubiquitous Sensor Networks (USN)

## Abstract

Web Portals function as a single point of access to information on the World Wide Web (WWW). The web portal always contacts the portal’s gateway for the information flow that causes network traffic over the Internet. Moreover, it provides real time/dynamic access to the stored information, but not access to the real time information. This inherent functionality of web portals limits their role for resource constrained digital devices in the Ubiquitous era (U-era). This paper presents a framework for the web portal in the U-era. We have introduced the concept of Local Regions in the proposed framework, so that the local queries could be solved locally rather than having to route them over the Internet. Moreover, our framework enables one-to-one device communication for real time information flow. To provide an in-depth analysis, firstly, we provide an analytical model for query processing at the servers for our framework-oriented web portal. At the end, we have deployed a testbed, as one of the world’s largest IP based wireless sensor networks testbed, and real time measurements are observed that prove the efficacy and workability of the proposed framework.

## 1. Introduction

The ubiquitous sensor network (USN) is drawing a lot of attention as a method for realizing a ubiquitous society. Through ubiquitous networks, a lot of information – not so important individually, is collected in large numbers to establish important information on a wider scope [1]. To make USN a reality in our daily life, the research community is paying its attention towards different branches of this next generation network. For instance, the performance of the network is widely examined from viewpoints of robustness, energy efficiency, scalability, etc. Whereas the hardware technologies such as MICA motes [2], Smart-its [3], and U^3^ [4] are proposed for the development of emerging applications. On the other hand, Sensor Web [5], Sensor Model Language (SensorML) [6], and IPv6 over Low power Personal Area Networks (6LoWPAN) [7] are the key standardization areas of USN. Moreover, a wide range of USN applications in health care, environmental monitoring, home automation, object localization, social participatory computing, and raw data to knowledge representation [8], etc., have emerged as roles of web portals in the U-era. The web portal will function as a single access point to all of the desired USN applications, as the current web portal does for the Internet applications. But, the current web portal architecture is not designed for resource constrained devices. Since it always communicates with the portal’s servers for each user query it makes, the resulting delays and high Internet traffic generated causes network congestion. The current architecture is also unable to provide access to real time information, because storing real time information on web servers means increasing the network traffic two-fold (i.e. pulling information from the network and storing it in the servers, and then entertaining the user query). Also storing of real time information, like humidity, temperature, etc. on servers is not only highly resource inefficient but also it’s not wise to do so. In other words, the information that has been reported to the servers but never used by the user brings unwanted communication and occupies unnecessary space in the servers. Hence there is a need for a framework that may broaden the role of the web portal in our daily life.

In this paper, we propose a framework for the web portal in the U-era. There are several factors which are to be considered while designing the framework: 1) It must provide access to the real time information: thus, we enable one-to-one device communication for providing quick access to the real time information. 2) Users must not be aware of the fact that the application interacts with a specific hardware platform: i.e. the proposed framework must provide abstraction to the lower layers. 3) Every digital device would be capable of communication, resulting into high traffic generation; thus the proposed framework must provide a built in network congestion control functionality: we distinguish the traffic types as global and local queries. Local queries are restricted within the local region of interest, and only global queries are sent over the Internet. We have also observed that there are two types of queries, i.e. specific and general. Specific queries address a specific audience, like nearest gas-station, and current temperature in Seoul etc; Whereas the general queries like stock exchange indexes, flight schedules, and latest news, etc. address a wider audience.

By keeping in mind all these requirements and facts of the U-era, we have proposed a framework for web portals. We have logically divided the framework into different local regions, where each local region only addresses a particular region. The main idea behind local region is that the queries which are meant for the same region, are solved locally rather than routed through the Internet. For the evaluation of our proposed framework, we firstly model the service response time at the servers by providing an analytical model; and then we have deployed a testbed that provides end-to-end analysis (combined effect of the network and the servers’ model) of the query processing. We have offered different applications on the single platform, and tested the feasibility of these applications. Through the testbed results, we have measured the performance of local regions, in terms of query response time, and the amount of total traffic generation. Moreover, we have compared the throughput of our framework with the receiver-centric congestion control mechanism of the web portal, as described in [9]. Our evaluation shows that the framework is well suited for all sensor applications with significantly low traffic overhead.

The rest of the paper is organized as follows. Section 2 describes the related work. The motivation is discussed in Section 3. We present web portal framework in Section 4, followed by the analytical and experimental performance evaluation in Sections 5 and 6 respectively. Section 7 concludes the paper, and describes the future work.

## 2. Related Work

Researchers have made various contributions by identifying potential research areas in a ubiquitous pervasive environment, and paying significant efforts to it. We can classify these into efforts with similar application goals (i.e. sensor web systems and sensor model languages, etc.) and those that have significant impact in our framework [i.e. service oriented architecture, ubiquitous service discovery, cluster-based Wireless Sensor Networks (WSNs), event servers role in WSNs, three-tier architecture for WSNs, and human centric search system, etc.].

**Sensor Web System:** The term “Sensor Web” is sometimes used to refer to sensors connected to the Internet. The purpose of a Sensor Web system is to extract knowledge from the data it collects, and use this information in order to react intelligently and to adapt according to its surroundings. It links a remote end user's cognizance with the observed environment [10]. A sensor web consists of a number of sensor platforms, also called pods, which can be fixed or mobile. Through pods, Sensor Web spreads the collected data and processed information throughout its entire network. By definition, a Sensor Web is an autonomous, stand-alone sensing entity that does not require the presence of the World Wide Web (WWW) to function [11].

**SensorML, a Sensor Model Language:** In today’s Internet, web contents are described through Hypertext Markup Language (HTML), which is a predominant markup language for web pages. Similarly, there is a need to present the U-contents in a standardized way over the Sensor Web. The Open Geospatial Consortium (OGC) describes *SensorML*, a language for moving sensor data among sensor nodes, and onto the Internet. The OGC SensorML specification defines it as a standard model and XML Schema for describing sensors systems and processes. It provides information needed for discovery of sensors, location of sensor observations, processing of low-level sensor observations, and listing of task-able properties [12].

**Service Oriented Architectures:** Web services approach for the design of sensor networks has been proposed in [13]. The purpose was to enable a flexible architecture where sensor networks data can be accessed worldwide. In this approach, sensor nodes are service providers, whereas applications are clients of those services. Sensor nodes publish their services by sending the services’ description to the sink nodes. The Web Services Description Language (WSDL) is used to describe data and functionalities of sensor nodes. Sink nodes provide the service descriptions of the whole sensor network. They act primarily as service providers to the external environment. Applications submit their service requests to the sink nodes which then interact with the appropriate sensor nodes requesting their specialized services that meet the user’s application needs.

**Ubiquitous Service Discovery:** Ubiquitous service discovery is not similar to the service discovery in WWW, like service discovery through JINI, UPnP, and SLP, etc. This is due to the fact that in ubiquitous environment, applications and services are not deployed onto a pre-existing network, but instead the network itself grows out of the applications and services the users want [14]. This approach enables users to view the network in the manner most appropriate to them and their requirements.

**Cluster-based WSN Applications:** WSN design dimensions include deployment, mobility, resources, cost, energy, heterogeneity, modality, infrastructure, topology, coverage, connectivity, size, lifetime and QoS. Example applications include Great Duck (bird observation on Great Duck island), ZebraNet, Glacier Monitoring, Cattle Herding, Bathymetry, Ocean Water Monitoring, Grape monitoring, Cold Chain Management, Rescue of Avalanche Victims, Vital Sign Monitoring, Power monitoring, Parts Assembly, Tracking Military Vehicles, and Self-healing Mine Field and Sniper Localization [15–17]. Our portal architecture is also influenced by cluster-based networks as by introducing the concept of local region in which sensor nodes are facilitated by the master nodes (whose role is similar as of cluster head).

**Role of Event Servers in WSNs:** A typical event detection mechanism in recent work on sensor databases is to set some thresholds for sensor readings in a query [18,19]. For example, when an object moves, the accelerometer attached to the object will report an increased acceleration reading. Based on this reasoning, an application program using thresholds will regard an event occurrence when the sensor readings exceed the pre-defined thresholds. Thresholds alone may be unable to fully specify an event for some applications. Thus, some approaches for event detection is based on the spatiotemporal patterns in sensor readings instead of simple thresholds. Since sensor networks are deployed in a physical space and sensor readings are collected over time, the changes in the sensor readings of networked nodes that are caused by an event usually exhibit some spatio-temporal pattern. This observation has been confirmed by the studies in various fields and analysis of real-world sensory datasets [20–23].

As the matter of fact, each device in U-era is expected to be capable of network communication, thus the process of reporting events to a central server and then pulling the event information by the user is inefficient. Due to the following issues, event based WSNs are not feasible in the U-era.

1. The events which are reported but never used by the user, so it is the waste of event reporting communication, and space in the servers.
2. Reporting events will cause a network overhead. Think of a scenario where a huge number of events could occur, so event reporting would manifold the network traffic. Moreover, if event reporting is made among different networks, via Internet; then it may choke the Internet.

**Modeling a Three-Tier Architecture for Sparse WSNs:** The traffic monitoring system MULE [24] is an example of a three-tier sensor network architecture that provides wide-area connectivity for a sparse sensor network by exploiting mobile agents such as people, animals, or vehicles moving in the environment. The top tier comprises wide area network (WAN) connected devices, the middle tier comprises mobile transport agents and the bottom tier comprises fixed wireless sensor nodes. Key traits of a MULE include large storage capacities (relative to sensors), renewable power, and the ability to communicate with the sensors and networked access points.

Also, SensEye [25] is a multi-tier network of heterogeneous wireless sensor camera nodes organized hierarchically across multiple tiers, unlike two-tier surveillance networks with low power cameras at the bottom tier that trigger higher resolution cameras at the upper tier. The multi-tier network achieves an order of magnitude reduction in energy consumption compared to single-tier networks, without sacrificing reliability.

MULE and SensEye provide an efficient architecture for traffic monitoring and surveillance respectively. But, they are not addressed as a generic architecture for U-applications, like U-health, U-shopping and U-information etc. In this paper, we assert the need of portal for sensor world, a light-weight, efficient and reliable architecture.

**Human-Centric Search System of the Physical World:** MAX [26] is a system for human-centric search of the physical world. MAX allows people to search and locate physical objects when they are needed. It provides location information reference to identifiable landmarks rather than precise coordinates. MAX was designed with the objectives of privacy, efficient search of a tagged object, and human-centric operation. MAX uses a hierarchical architecture that requires objects to be tagged, sub-stations as landmarks, and base-station computers to locate the object. Tags on objects can be marked as private or public which is searchable by the public or the owner only.

Applying and removing tags in the U-era is cumbersome and may bring the inconsistencies with the system. Moreover, there could be a huge variety of tagging options, like private, public, groups, restricted etc. that are not only difficult to manage but may also lead to security vulnerabilities.

## 3. Motivation

The U-era can be referred to as a fourth generation age where application requirements, information type and services are different from those that are available in the Internet age. U-applications enable dynamic access to real time information of interest, rather than presenting the static information stored in the servers. The most popular U-applications are U-health, U-monitoring, and U-traffic, etc. U-health refers to a system which provides appropriate advice and suggestions to the patient in a comprehensive manner according to the situation. Similarly, U-monitoring and U-traffic systems monitor their environments and inform customers about important events, and give suggestions comprehensively. These U-applications are constantly relying upon the real time information and involve the monitoring of the environment on a very regular basis. By keeping the requirements in view, we need a framework over the web portal architecture which provides a point of access to the real time information. Moreover, the development in the Sensor Web system also urges us to provide a framework that broadens the scope of the web portal architecture by making it accessible over the Sensor Web or the Internet.

### 3.1. Why not the Current Web Portal Architecture?

There are two types of model that can be deployed over web portals, i.e. sender-push, and receiver-pull [27]. In the sender-push model, a sender can deliver traffic at will to a receiver, who can only passively accept the traffic, such as in the SMTP-based email delivery system. In contrast, in the receiver-pull model, the receivers can regulate if and when they wish to retrieve data, such as the HTTP-based web access system [27]. We argue that the problem of unwanted Internet traffic can be mitigated to a great extent if the receiver-pull model is employed by Internet applications in the U-era, whenever appropriate. Because in order to serve the users’ queries, the web servers need to store the real time information, like temperature, traffic and humidity information etc. — that means a huge amount of information is being periodically stored on the servers. Then the user would request some query and those servers would reply after searching from their pool of databases. This implementation is unlikely to be acceptable in the U-era; because it not only manifolds the Internet traffic, but also storing a huge amount of information, that may never be requested, is meaningless. Thus, web portal architecture is designed to provide real time access to the stored information, but not access to the real time information. Moreover, it’s strong client-server architecture that may not be suitable for quick flow of information in a U-environment. Similarly, our testbed results (as described in Section 6) show a high end-to-end delay for general web portal architecture as compared to our framework-oriented web portal, and Internet routers’ inability to handle a huge number of packets also support our observation that the current web portal implementation for U-era is not feasible.

As a solution, one-to-one device communication over the network provides quicker access to the real time information, with little overhead. Our testbed results (as described in Section 6) show that one-to-one device communication is up to three times faster than that of conventional web portals. Whereas the overhead caused by one-to-one device communication can be categorized into: i) communication overhead: i.e. the network overhead which is a major factor for an increased en-to-end delay; and ii) application layer protocol overhead: like HTTP overhead which is mainly caused by HTTP and TCP interactions [28]. The architectural difference between the current web portal and the proposed framework-oriented web portal is explained in Section 4.1.

### 3.2. The Framework and Its Desirable Features

By keeping in view the requirements in the U-era, we have thought about the powerful framework platform that would serve the same purpose as the web portal has in the Internet age. The framework is designed by considering the following key requirements:

- It must address an easy access to the real time information.
- It must be able to offer a huge *portal space* i.e. a variety of U-applications could be offered over a single portal application.
- It must address all kinds of sensors networks, i.e. homogeneous and heterogeneous sensor networks.

## 4. An Architectural Framework of Web Portal in Pervasive Environment

**Figure 1.**
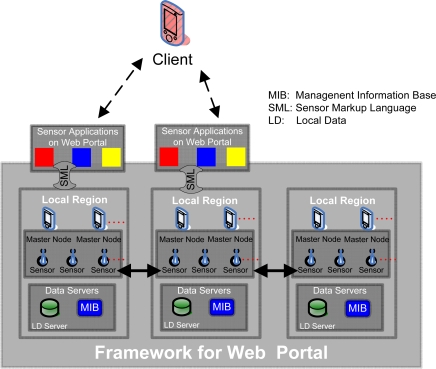
An architectural framework for a web portal in a pervasive environment.

In accordance with the sensor requirements and issues, we have proposed a web portal framework architecture for pervasive environment, which is hybrid in nature. The framework broadens the role of web portal and enables its importance in the pervasive environment. Figure 1 explains the architectural framework:

**Sensor Applications:** Web Portal is a platform where multiple sensor applications, like U-traffic, U-health, U-management etc. could be provided. Sensor applications communicate with the sensors through a standard language, like SensorML.

**Client:** Client can access various sensor applications that are built upon the web portal, like temperature information, traffic information, and to a particular object information etc. Now, we can logically divide the whole region into different local regions.

**Local Regions Layer**: This layer serves as the backbone of the whole framework. This layer can be divided into a number of local region(s).

**Local Regions:** In the U-environment, each device is equipped with computation and communication abilities. For better information management and overall traffic reduction, a number of local region tiers are introduced. In fact each local region tier represents a particular region like a city or a town. We believe that the most of the user’s queries would be pertaining to the region to which he/she belongs, at the time of querying. The user could also be willing to know the current temperature, best possible traffic route, the hottest deal available on a particular product offered by the nearby store, emergency notification to the nearest concerned office etc. We assert that each local region represents a particular region, where regional queries would be solved within the region rather than contacting the global server/information hub that causes the latency and traffic congestion.

**Master Node:** A master node is a relatively powerful sensor node compared to the other sensor nodes in the network. It acts like a central authority which manages all the information to and from the sensor nodes that are associated with the master node. Thus, the basic motivation of introducing the master node is to reduce the computational and the communication burden from the sensor nodes; because the sensor nodes are low power and low processing devices, and would not be able to compute each time queried. Moreover, in order to resolve the query, multiple sensors collaborate with each other and a final result is replied to the user, this all management is not possible with just a couple of sensor nodes.

**Data Servers:** The Data Severs include Local Data (LD) Server, and Management Information Base (MIB) Server. The **Local Data (LD) Server** keeps the information and the data pertaining to the region, for which they are deployed for; whereas the **MIB Server** keeps the management-related information for the sensor nodes deployed in that region.

**The Global Servers Layer**: These global servers are part of current web portal architecture. Here we want to highlight the role of these servers in a pervasive environment. Data Servers used in the current web portal architecture are also referred to as Global Data (GD) Servers that provide the global information. At this point, it is very important to distinguish between LD and GD. LD means the data which is for the interest of local users, like the directions towards the nearest gas-station, or the current temperature in the local region, etc., whereas the GD is of interest to a larger community and these queries are independent of the object’s location. For example, stock exchange information, flight information, and the latest news updates are part of GD and are stored in GD servers. These applications may be queried by any user, or from any local region.

Clients can access the various sensor applications. These sensor applications connect with the appropriate local region; and then the master node, with the help of its associated sensor nodes, tries to resolve that query. If the query is resolved within the local region then the result is replied back to the client node. If the query could not be resolved within the local region itself, then the master nodes try to resolve the query by involving Global Servers (of web portal) and/or other local region (s).

### 4.1. An Architectural Difference, Current Web Portal and Proposed Framework-Oriented Web Portal

**Figure 2.**
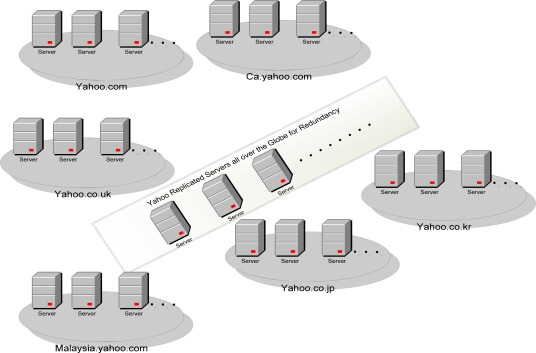
Yahoo regional and replicated servers for the Yahoo portal.

In order to meet the regional queries and reduce the network latency, different tailor-made web portal sites are developed and deployed in that region/country. For example, as in Figure 2, Yahoo has provided different web portals for different regions. These portals target the audience of that particular region. Moreover, for redundancy and load balancing among the servers, the mirrored servers are also installed at different locations [29].

The web portal architecture is not developed for providing access to real time information like current temperature and humidity information at a particular region. If we implement “pull architecture” onto the servers, as used by the web portal servers, then the servers not only need to store a huge amount of information, but this also results in a lot of network traffic. At the other hand, the proposed framework is well capable of addressing the huge requests and able to produce less traffic as well. Because the query is replied by the concerned sensor nodes, hence there is no need to store the raw information.

Moreover, current web portals’ architecture is built for high-end machines, which are capable of operating at high transmission speeds, i.e. more than 1Gbits/sec, whereas in the U-era, the target hardware is often wireless and low powered, operatng at much lower transmission speeds, i.e. up to 256 Kbps. Thus, high-end machines, which are unaware of receiver’s transmission capability, may cause traffic loss at low transmission operated receivers, i.e. sensor nodes. The proposed architecture handles this key requirement efficiently by introducing multiple master nodes in each local region. These master nodes, which are operating in the low-speed network, are capable of managing the packets to/from low-speed and high speed networks.

### 4.2. Client’s Interaction over the Web Portal with and without Proposed Framework

In this section, we diagrammatically describe the different ways of client interaction over the web portal. We have shown that the client may access the web portal through sensor networks (i.e. using IEEE802.15.4 technology), through Wi-Fi networks (i.e. IEEE802.11 technology), and through our proposed framework.

**Figure 3.**
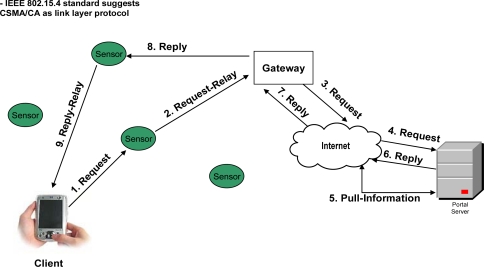
Client interaction over a web portal through sensor networks in general.

**Figure 4.**
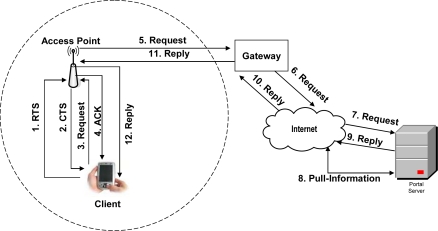
Client interaction over a web portal through Wi-Fi.

**Figure 5.**
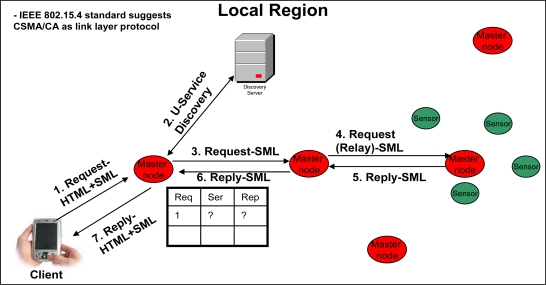
Client interaction over the proposed framework (within local region).

**Figure 6.**
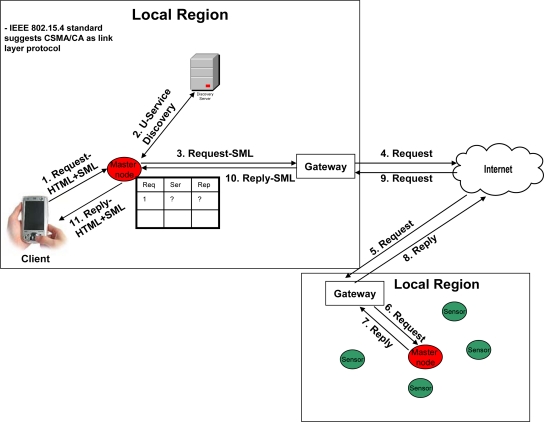
Client interaction over the proposed framework (outside local region).

Figures 3–6 elaborate the possible ways of a client’s interaction over a web portal with and without proposed framework. In the case of current web portal architecture only, there are two possible ways of interaction of clients with the web portal. As shown in Figure 3, A) *Client’s Interaction through sensor networks in general*: The client that wants to make a query request, first listens to the network. If the network is busy, the transmission is held off until later.

On the other hand, if the medium remains free for a certain period of time (called DIFS for Distributed Inter-frame Space), then the client can transmit the signals (make the request) to its nearest sensor node. In other words, we can say that both client and sensor nodes use CSMA/CA as link layer protocol [30], as defined by the IEEE 802.15.4 standard [30]. Client’s request is handled by the sensor node that will forward the request to the gateway (which then connects the internal network to the Internet). Once the gateway receives the request, then it connects with the web portal’s server in order to get the response.

In Figure 4, B) *Client may access the portal through Wi-Fi*: In case of Wi-Fi [31], once the client finds the medium as free, then it transmits a Ready to Send (RTS) packet containing information on the amount of data (i.e. request) that it wishes to send, and its transmission speed. The receiver (generally an Access Point (AP)) responds with a Clear to Send (CTS) packet; and then the client makes query request. When query request data sent by the client (i.e. client finishes its query request) has been received, the receiver sends an acknowledgement notice (ACK) to the client. In other words, we can say that Wi-Fi uses CSMA/CA as underlying link layer protocol – supplemented by the exchange of RTS and CTS packets among the client and AP. At the other hand, when AP receives the request, it contacts the web portal’s server through the Internet in order to get the response, and then replies the client.

As discussed earlier, in U-environment the most of the information would be real time information, thus the portal servers need to implement pull architecture in order to get the information from the external entity. This communication introduces a huge traffic over the Internet. Moreover, the implementation of Wi-Fi in the U-era may provide the wireless access to the Internet, i.e. the concept of providing access to the web portal at anywhere and anytime. But, Wi-Fi cannot address the limitations of web portal in the U-era, as discussed in Section 3.1. At the other hand, in wireless sensor networks, the sensor nodes are not only capable of wireless communication but also cooperatively monitor physical or environmental conditions, such as temperature, sound, vibration, pressure, motion or pollutants, at different locations etc. Whereas enabling IPv6 over the sensor nodes make them accessible anywhere and anytime. Thus, these sensor nodes have not only the capability of network communication but also observe the real time information, which make them suitable technology over Wi-Fi for the flow of real time information.

Figures 5 and 6 best describe a client’s interaction scenario in our framework-oriented web portal architecture. A client makes a request to its immediate master node, which keeps the entry in its table. That master node discovers the service from the discovery server and directly contacts the target master node (that may be within the same local region, as shown in Figure 5, or within some different local region, as shown in Figure 6) that can resolve the query. On the reply from the query, the requested master node replies to the client. Note that due to the heterogeneous nature of the sensor network, the communication between the sensor nodes is over SML. Moreover, as described above in “Client’s Interaction through sensor networks in general”, the client, master nodes and the senor nodes use CSMA/CA as link layer protocol [30], as defined by the IEEE 802.15.4 standard [30], in our approach.

## 5. Analytical Evaluation of the Query Processing at the Servers

In this section, we provide an analytical model for the query processing at the servers for our framework-oriented web portal; whereas the network model has been studied through the testbed results, as in Section 6. There are various types of servers that have been introduced in our framework, i.e. Service Discovery servers, LD servers, MIB servers, and GD servers etc. Thus, it is important to provide an analysis of query processing at these servers for our proposed framework. This analysis can be studied through an analytical evaluation, as its best.

We have benchmarked the web portal’s multi-tier architectural design for query processing at different servers for our framework. For web portal application, a single request is served by hundreds of applications operating in parallel [32]. Such elementary software applications, designed to be composed with each other, are commonly referred to as *services*. The data servers’ tier of our proposed framework and Global Servers tier of web portal are logically modeled into the business logic tier, and the servers tier. For fast query response, the business logic code generates multiple queries to its underlying databases (servers tier) to generate a single response.

**Figure 7.**
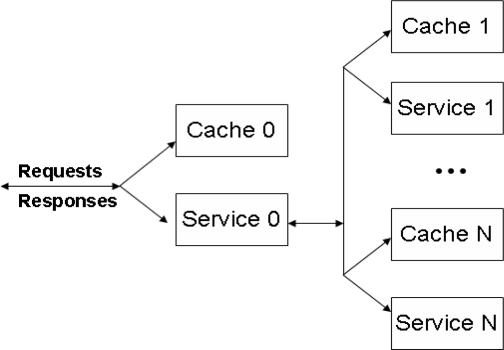
Analytical model for multi-tiered web applications.

There are various analysis models proposed for multi-tier web architecture that can be applied in our proposed framework-oriented web portal architecture, but the model presented in [33] best describes the approach. As described in [33], the query requests arrive according to a Poisson process with rate *λ* to a dedicated entry level 0 (where all requests arrive). If the query response is cached at caching tier 0 with probability *P0*, then the response will be made to the client, without any further processing. However, the business logic tier will execute the query, if the query needs to be processed with probability *1−P0*, by service tier 0 and the other *N* levels in the queuing network, as depicted in Figure 7. The request is routed to each of the *N* levels in sequence, where at each level *i*, the request is served by caching tier *i* with probability *P_i_*. If the request is not cached at the caching tier, with probability *1* − *P_i_*, the request is served by service tier *i*. When the query is solved at level *i*, the results are sent back for processing to service tier 0. For fast query processing, a request that is sent from the business logic to each level *i* generates *K_i_* requests back and forth, where *K_i_* is a non-negative discrete random variable. Thus, a request has fully completed its service at level *i* after *K_i_* service completions. Hence, every request that is served by service tier 0 passes this tier 1 + *K_1_* + *K_2_*· · · + *K_N_* times, and finally leaves the system after having visited all *N* levels.

Each caching tier *i* is modeled by an infinite-server queue having general service times with an average of *γ_c,i_* time units for *i* = 0,...,*N*. Whereas, each service tier *i* is modeled by a processing sharing queue with an average service time of *γ_s,i_* time units for *i* = 0,...,*N*. The mean response time of the service can be obtained through the sojourn time of the request in the system. Let *S_i_^(k)^* be the sojourn time of the *k-th* visit to level *i*, and *M* = *EK_1_* +· · · + *EK_N_*. Then, the expected sojourn time *ES* of an arbitrary request arriving to the system is given by:

$$\mathrm{ES}=E\left[\sum_{k=1}^{M+1}\;S_{0}{}^{\left(k\right)}+\sum_{i=1}^{N}\;\sum_{j=1}^{K_{i}}\;S_{i}{}^{\left(j\right)}\right]$$

Now, we say that *L_c,i_* and *L_s,i_* denote the stationary number of requests at caching tier *i,* and service tier *i* for *i* = 1,...,*N*, respectively, we have:

$$\begin{array}{l} X(L_{c,i}=l_{c,i},\;L_{s,i}=l_{s,i};i=0,...,N)=\\ \prod_{i=0}^{N}\;X(L_{c,i}=l_{c,i})\;\prod_{i=0}^{N}\;X(L_{s,i}=l_{s,i}) \end{array}$$

with *l_c,i_* and *l_s,i_* = 0, 1,... for *i* = 0,...,*N*. We can also determine the expected sojourn time at the entry node and the service nodes. Let *ρ_c,0_* = *λ_P0_γ_c,0_* defines the load on the entry node’s caching tier, and *ρ_s,0_* = (*M* + 1)(1 − *P_0_*)*λγ_s,0_* for the service tier. Similarly, the load for the basic services is given by *ρ_c,i_* = (1 − *P_0_*)*P_i_λγ_c,i_* and *ρ_s,i_* = (1 − *P_0_*)(1 − *P_i_*)*λγ_s,i_* for *i* = 1,...,*N*. Then, by Little’s Law, the expected sojourn time *ES*_c,i_ at caching tier *i* is given by *ES_c,i_* = *γ_c,i_*, whereas the expected sojourn time *ES_s,i_* at service tier *i* is given by *ES_s,i_* = *γ_s,i_*/(1 − *ρ_s,i_*) for *i* = 0,...,*N*. Combining all the expressions for the expected sojourn time at each tier in the network, we derive that the expected response time is given by:

$$\begin{array}{l} \mathrm{ES}=E\left[\sum_{k=1}^{M+1}\;S_{0}{}^{\left(k\right)}+\sum_{i=1}^{N}\;\sum_{j=1}^{K_{i}}\;S_{i}{}^{\left(j\right)}\right]=P_{0}\;{\mathrm{ES}}_{c,0}+\\ (1-P_{0})\left[{\mathrm{ES}}_{s,0}+\sum_{i=1}^{N}\;{\mathrm{EK}}_{i}\{P_{i}\;{\mathrm{ES}}_{c,i}+(1-P_{i})\;{\mathrm{ES}}_{s,i}\}\right]\\ =P_{0}\;\gamma_{c,0}+(1-P_{0})\;\frac{(M+1)\;\gamma_{s,0}}{1-\rho_{s,0}}+\\ (1-P_{0})\;\sum_{i=0}^{N}\;{\mathrm{EK}}_{i}\left[P_{i}\;\gamma_{c,i}+(1-P_{i})\;\frac{\gamma_{s,i}}{1-\rho_{s,i}}\right] \end{array}$$

## 6. Experimental Evaluation of the Proposed Framework

We have evaluated the proposed architecture based on three important aspects: i) total network delay with and without local region, ii) the behavior of the network, with and without framework-oriented web portal, during a heavy traffic, and iii) throughput under end-to-end delay for our proposed framework and receiver-oriented congestion control mechanism used by web portals [9].

### 6.1. Network Model of the Testbed

**Figure 8.**
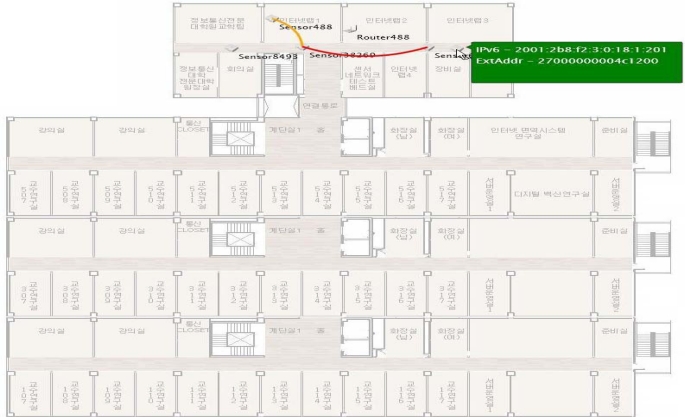
Network topology of the testbed deployed.

We examined the proposed framework architecture by deploying a testbed of 150 sensor nodes, in 1^st^, 3^rd^ and 5^th^ floor of eight-story building of Ajou University. We have used temperature sensors, heat sensors, humidity sensors, and sound sensors, for framework evaluation. Each sensor node contains Chipcon’s CC2420DB IEEE 802.15.4 compliant wireless mote. Each mote is assigned with an IPv6 address, and the total 50 nodes are installed in the rooms and corridors of each floor, with an average distance of 3 meters. Each Sensor mote is part of a PAN (Personal Area Networks), and there are total 5 PANs at each floor. There is one PAN coordinator within each PAN. The PAN coordinator is a powerful sensor node that is main powered, and acts as a master node. Figure 8 shows the network topology of the testbed deployed.

We have developed the portal application by using our own software platform (i.e. protocol stack) which is developed in C programming language, named as Stack6. Stack6 is proprietary of our lab and enables the IEEE802.15.4 communication using the TCP/IPv6 protocol. We have considered each floor of the building as a local region. Each local region is connected with the sensor gateway, which regulates the traffic to-and-from the local regions. To evaluate the web portal’s performance without the local region, we have connected the web portal application with the external router. Now the query can either be processed through local region or without local region.

### 6.2. Experimental Results

**Figure 9.**
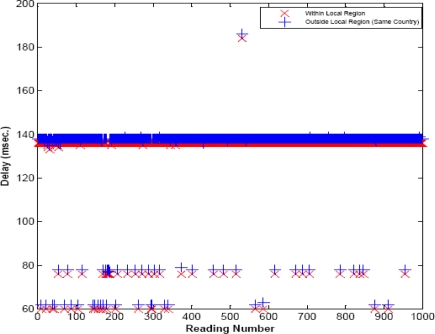
End-to-end delay when query is resolved within local region or over the Internet (same region).

**Figure 10.**
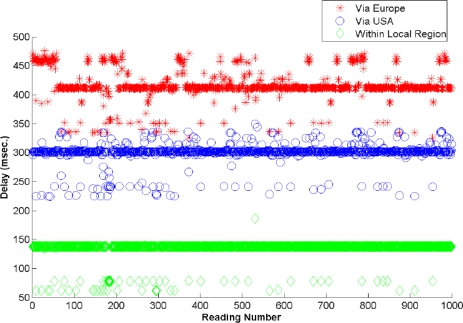
End-to-end delay when query is resolved within local region or over the Internet (via Europe and via USA).

To evaluate the efficacy of the proposed framework, we have performed more than 1,000 experiments to calculate the total end-to-end query response time, and the total network traffic overhead, with and without local region. Figures 9 and 10 show the average end-to-end query response time, i.e. query processing time at the servers (as modeled in Section 5) and the network delay time. Note that the sensors in local region can handle maximum traffic of 256 kbps. To route the packets over the Internet, we have used loose source routing options in the IPv6 header format. Moreover, we have seen that the round trip time varies region to region.

In Figure 9, the average delay of 2 ms is observed when the query is resolved within the local region or within the same country (where the query is made), i.e. Korea. The delay within the local region is very smooth (almost constant), but different delay values are observed as shown in Figures 9 and 10. This is due to the reason that the query can be cached within the master node, which cause the minimum delay; whereas it usually requires two master nodes for processing the query, causing the delay of almost 136 ms. If the immediate master node, from the client, is busy then more than two master nodes process the query, that adds more delays. At the other hand, as shown in Figure 10, a huge delay can be seen from 150 ms to 450 ms, when the query is routed via USA or Europe. It can be observed from Figures 9 and 10 that the query response time can be reduced when the query is resolved within the local region. Whereas routing the packet over the Internet adds processing, and queuing delay at each router of the Internet. If the service discovery time is added, then the query response time would dramatically increase in case of general web portal architecture; whereas in local region it would not affect a lot.

**Figure 11.**
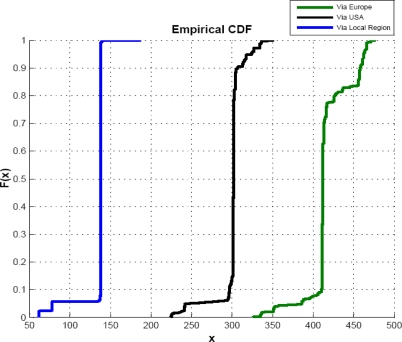
CDF of end-to-end delay analysis.

There are several factors, like link failure, change in routing path etc., which cause the network delay; but the network load is usually the main reason that causes fluctuation in the end-to-end delay and, therefore, can be thought as one of the most important reasons among all the factors that cause fluctuation in delay [34]. Figure 11 shows the cumulative distribution function (CDF) of end-to-end delay of Figure 11. We can observe from Figure 11, that different regions have different CDF values at the given point. We can observe the CDF graph of “within local region” is almost smooth, showing the balanced network load. Delays can indicate the network load situation very well based on the analysis of the CDF of end-to-end delay in a given path.

#### Congestion Control Comparison between Web Portal and Proposed Framework

Current web portal architecture exploits the information available only at receivers in order to improve latency and throughput in diverse scenarios of portal application. In other words, TCP protocols delegate key congestion control functions to the receivers [9]. Whereas in our proposed framework i) the local regions restrict the traffic within their proximity, hence avoiding the congestion to increase, ii) furthermore, the congestion is normalized among the multiple master nodes within the local region.

**Figure 12.**
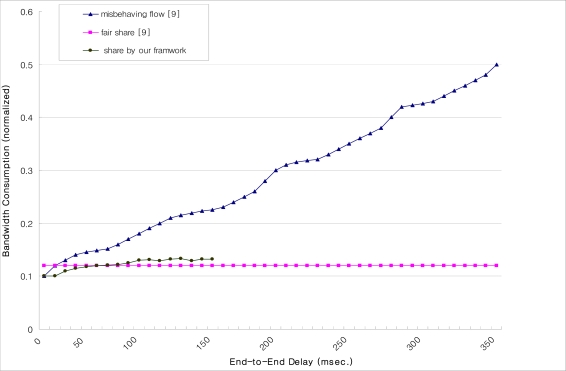
Throughput comparison with the delay.

We have analytically compared the results obtained by Receiver-centric congestion control mechanism, described in [9], and with our proposed mechanism. In [9] the bandwidth consumption has been computed using the square-root TCP-friendly formula [35]. Thus, in order to provide a fair comparison, we have obtained our results with the same formula, as in [35]. In Figure 12, we plot the bandwidth of a single flow (on the y-axis) against variable end-to-end delay of the flows from 20 to 350 ms (as shown on the x-axis). It is observed that when all flows are well-behaved, the bandwidth share is fair [9] (the straight line in the figure). However, as the flows of end-to-end delay increase, the malicious flow steals more and more bandwidth, up to five times more than its fair share (the maximum for this scenario) when the end-to-end delay is 350 ms [9].

But, in case of our proposed framework, the max end-to-end delay is observed to be 150 ms, and mostly it lies between 50 ms to 100 ms. Thus, the bandwidth occupied in our proposed framework is closer to the well behaved flows of the web portal (which is the ideal case of web portal). Also, at the end-to-end delay of 150 ms, our framework consumes half of the bandwidth, i.e. an improvement of 50%, compared to the misbehaving flow. But, as shown, increasing end-to-end delay of misbehaving flow causes more bandwidth consumption – thus, at one stage, our scheme proves to be up to five times better than the misbehaving flow.

**Figure 13.**
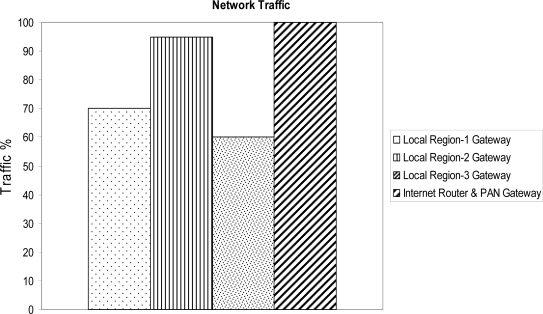
Heavy traffic at network gateways/router.

In our experiments, we have also created a U-era scenario by generating a huge number of packets and injected them, from each floor, into the network with an interval of less than 1 ms. There are two types of packets that have been injected into the network; 1^st^ ones that would be handled by the Internet router (as is always in the case of a web portal), and 2^nd^ those that would be handled within local region or by the local region gateway. As mentioned before, we assert that in U-era the most of the applications would generate the traffic that is within the local scope of the application, such applications could be home automation, object tracking, shopping mall applications etc. To observe the network behavior in our assertion, we have intentionally generated 40% or fewer packets for each floor (local region) – where the source and destination addresses are within the same local region. These packets will not be forwarded to the gateway and would be handled locally. It can be seen from Figure 13 that the Internet router has started dropping the packets, whereas the local region gateway is still be able to handle the traffic. This behavior is resulted due to the fact that the query packets, which are destined to the same local region, are handled locally; and are not forwarded to the local region gateway, resulting less congestion on local region gateway as compared to the Internet router. Moreover, both Internet and sensor networks work at different network bandwidth speed. In our scenario, sensors network bandwidth is 256 kbps; whereas the Internet bandwidth is up to 1 Gbps. There is a gateway that regulates the traffic between two different networks, known as PAN gateway. If the PAN gateway receives the packet with the speed more than that it can handle, then it also start dropping the packets as well.

## 7. Conclusions

The U-era enables the communication environment in which any informational service is accessible to anyone through any digital deice and network without any limitations of space and time. A variety of services would be offered in the U-era that require changes in the current web portal architecture. We have described in details that the current web portal architecture is not feasible in the U-era due to the nature of the query, which is real time; and also that may not require interaction with the database as well. It can also be observed that in the U-era most queries would concern the same region where the queries are made. In this paper, we have proposed an architectural framework for the web portal in a pervasive environment, and introduce the concept of local region. We have shown that through the concept of local region not only the query response time is improved but also the network overhead is reduced as well. We have evaluated our framework through one of the world’s largest IP based WSN testbed. We have observed that the web portal’s implementation with our proposed framework achieves the desired results and enhances the web portals requirement in U-era.

### 7.1. Future Work

As for our future work, we would verify the importance of MIB server, after deploying a large number of sensor nodes, and would discuss the management of sensor nodes. As the aim of this paper is to introduce web portal’s importance in the U-era, its concept and the architecture, i.e. framework for web portal; so our next step is to discuss the results from management of large number of sensors point of view.

## Figures and Tables

**A1.**
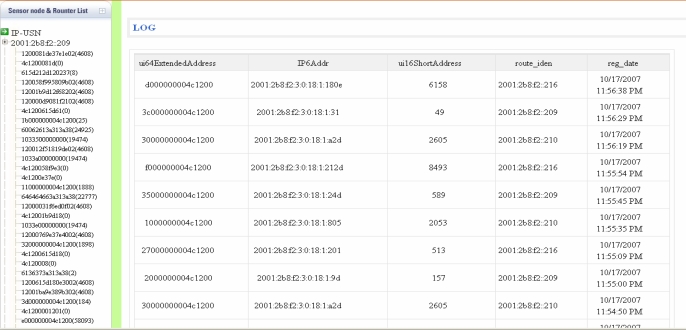
The Log file for the experiments.

**A2.**
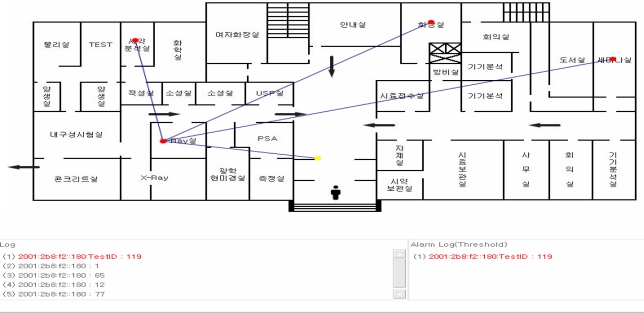
The message passing among the sensor node.

**A3.**
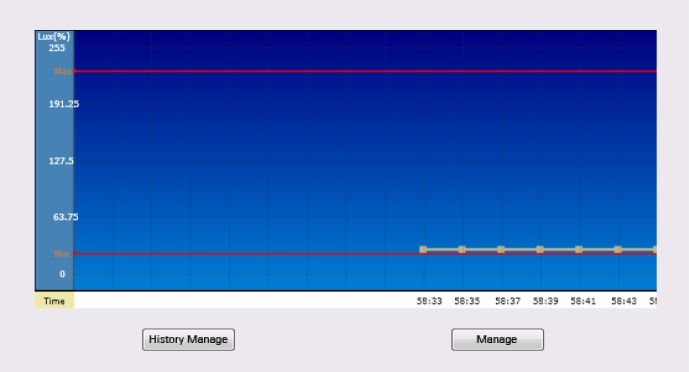
The graph created from the sensor data.
